# Emerging vaccine manufacturers are innovating for the next decade

**DOI:** 10.1016/j.jvacx.2020.100066

**Published:** 2020-04-29

**Authors:** Benoit Hayman, Sonia Pagliusi

**Affiliations:** DCVMN International, Route de Crassier 7, 1262 Nyon, Switzerland

**Keywords:** Vaccine manufacture, Vaccination, Polio eradication, Infectious diseases, Vaccine’s supply

## Abstract

The Developing Countries Vaccine Manufacturers Network (DCVMN) is a public health-driven alliance consisting of 43 vaccine manufacturers from 14 countries and territories, operating under the mandate to protect all people against known and emerging infectious diseases, by improving the availability of high-quality vaccines globally. The Network provides a platform for organizations to come together regularly to share technical information, best practices and future prospects. DCVMN members are playing an increasingly important role in public health supplying over 50% of the doses of vaccines procured by UNICEF globally. To evaluate the progress made by Network’s members, a survey consisting of 9 questions covering three important components of the manufacturers in the network was created, focusing on company dedicated human resources, vaccine production, and research and development efforts. Results show that more vaccines from more manufacturers are achieving WHO Pre-qualification, with areas of focus including the contributions to a Polio-free world, ending cholera, and tackling re-emerging diseases, such as measles. An increase by 50% of number of manufacturing companies holding WHO prequalified vaccines was observed from 2013 to 2019, strengthening open competitiveness for global vaccines supply. Notably, Network members have 181 vaccine projects in the research and development pipeline, highlighting novel vaccines against mosquito-borne diseases, such as dengue, chikungunya and Zika, novel human papillomavirus and pneumococcal conjugated vaccines. This report summarizes the progressive efforts of DCVMN members to contribute to reducing the burden of infectious diseases globally and details their commitment to vaccine innovation, particularly in the past five years, in the context of how vaccine innovations of today will shape the fight against infectious diseases tomorrow.

## Highlights

• DCVMN members collectively provide over 50% of vaccines supplied to UNICEF.
• Emerging manufacturers raised their WHO Prequalified vaccines number to over 70.
• 181 vaccine projects are reportedly in DCVMN members’ R&D pipelines.
• Product innovation and competitiveness will drive the future vaccine supply.

## 1 Introduction

The Developing Countries Vaccine Manufacturers Network (DCVMN) is a public health-driven alliance of corporate vaccine manufacturers based in developing countries, as defined by the United Nations’ World Economic Situation and Prospect report [1]. The Network operates under the mandate to protect all people against known and emerging infectious diseases, by improving the availability of high-quality vaccines globally, recognizing the need for international scientific, technical and economic cooperation.^1^ The Network was formed in the year 2000 by ten vaccine manufacturers [2], motivated by the World Health Organization and the Gavi Alliance, aiming to improve vaccine supply to developing countries where populations were growing rapidly, incidence of disease was high and purchasing power was low.

The Network provides a platform for organizations to come together regularly to share technical information, best practices and future prospects. This voluntary cooperation increases the pool of resources available to each respective member and also provides the opportunity for technology transfer, co-development and supply of products, as appropriate. This collaborative model improves manufacturers’ capabilities to supply affordable vaccines globally.

**Fig. 1:**
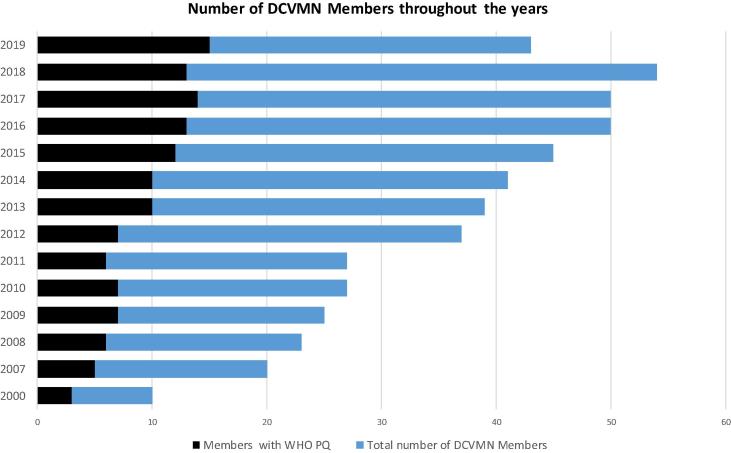
Number of DCVMN members starting in the year 2000 with ten members. Bar chart depicts the number of corporate Network members per year, from 2000 to 2019. The blue columns indicate the total number of DCVMN members. The black columns indicate how many of these members have a vaccine Pre-qualified by WHO. A Network milestone of 50 members was achieved in 2016. A slight decrease in membership occurred due to geo-political shifts and budget constraints resulted from an increase in membership fees in 2019. Data on the number of members between 2001 and 2006 was unavailable. (For interpretation of the references to colour in this figure legend, the reader is referred to the web version of this article.)

Since its inception the Network showed a steady growth achieving the milestone of 50 corporate members in 2016 (Fig. 1) and the vaccine and global health landscape has changed considerably. Notably, the number of manufacturers achieving WHO prequalification for their vaccines increased steadily, peaking at 15 in 2019. Increases in vaccine production, global supply and coverage helped reduce vaccine preventable deaths by 42% between 2000 and 2017 [3]; yet the incidence of known and emerging infectious diseases remains high. Inefficiencies in vaccine production, regulation and supply chain prevented global vaccine coverage from increasing, and technological advancements and innovations, such as combination vaccines, e.g. hexavalent vaccines, are required to fight infectious diseases, particularly in developing countries, where the majority of people are affected. As a unique international alliance of public and private organisations DCVMN is at the frontier to help solve the challenges the emerging vaccine industry faces.

As of December 2019, the Network included 43 corporate members from 14 countries and territories, based in Asia but also in Middle East, Africa and Latin America (Fig. 2a). These manufacturers were responsible then for employing over 80,000 people (Fig. 2b) increasing the welfare of families and communities in developing countries. Additionally, by building facilities and operating in developing countries, manufacturers strengthen their national economies and provide long-term career prospects. Research and policy organizations from all over the world that support the network can join as resource members.^2^

**Fig. 2a:**
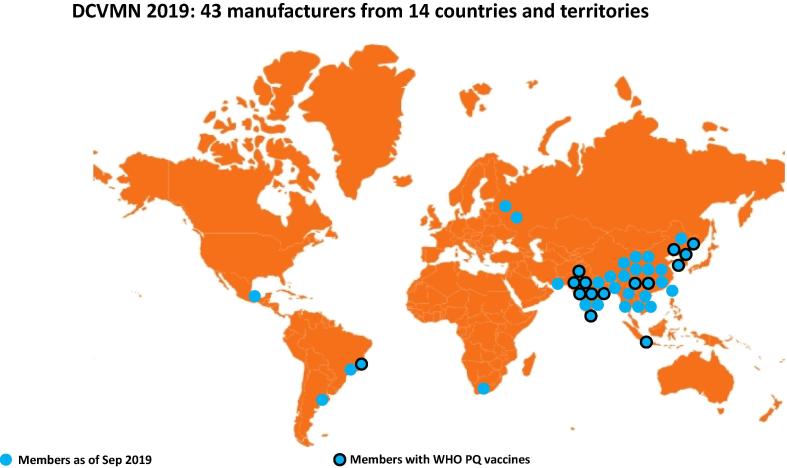
Geographical distribution of 43 DCVMN members globally. Geographical distribution of 43 DCVMN members in 14 countries and territories: Argentina, Bangladesh, Brazil, China, India, Indonesia, Mexico, Pakistan, Republic of Korea, Russia, South Africa, Taiwan, Thailand, Vietnam. As of October 2019, 15 members (indicated with dark-rim circles) have vaccine products that have been pre-qualified by the World Health Organisation.

**Fig. 2b:**
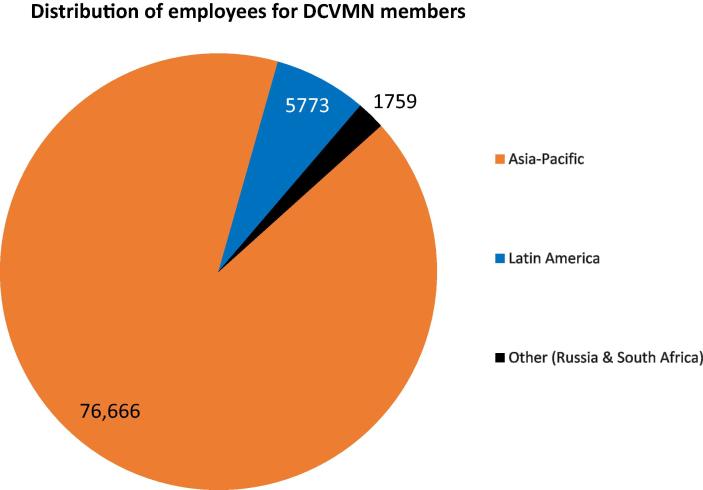
Regional distribution of direct employment provided by DCVMN corporate members. DCVMN members employed more than 80,000 workers globally: 76,666 reside in the Asia-Pacific region, 5773 in Latin America, and 1759 between Russia & South Africa (Other). Employment data, from a DCVMN survey conducted in June 2019, comes from 34 of the 43 manufacturers in the Network, representing approximately 80% of the Network.

In 2018, DCVMs supplied over 50% of the 2.36 billion doses of vaccines procured by UNICEF globally, valued US$1.453 billion [4] and contributed to GAVI-financed vaccines supplied in 2018. Based on the third dose of DTP (diphtheria-tetanus-pertussis) as key indicator, global infant vaccination has now reached 85% coverage [5], with pockets of low coverage causing 19.4 million children to be vulnerable to vaccine preventable diseases.^3^

In 2019, fifteen of the 43 Network corporate members held WHO pre-qualification - compared to seven in 2012 - for the vaccines produced respectively which are procured for immunization programmes all over the world, significantly contributing to improving global vaccine coverage. Recently multiple WHO regions have experienced large measles and diphtheria outbreaks, increasing the incidence of both diseases between 2017 and 2018.^4^ Of the under vaccinated children, 60% reside in 10 countries [6], therefore greater focus must be given to the development of vaccine presentations that optimize their affordability and programmatic ease of use in countries where under-vaccinated children reside, such as thermostable vaccines that do not require cold-chain. Since 2015, the Network established experts’ working groups to foster regulatory convergence [7], supply chain efficiencies and safety monitoring through pharmacovigilance, and is actively working on innovative strategies to help solve problems in these areas by accelerating access to needed vaccines, alongside international organizations such as WHO, UNICEF, PAHO and GAVI.

This report outlines the progressive efforts of DCVMN members to contribute to reducing the burden of infectious disease globally and details their commitment to vaccine innovation. The findings detailed here are based on a recent survey of all 43 members which focused on vaccine research and development, complemented with literature from multiple sources, illustrating the contributions by Network corporate members. The progress made, particularly in the past five years, is discussed in the context of how vaccine innovation today will shape the fight against infectious diseases tomorrow.

## 2 Methodology

To better understand the progress made by Network manufacturers, a survey consisting of 9 questions covering three important components of the 43 manufacturers in the network was created. The survey focused on company dedicated human resources, vaccine production, and research and development efforts. The questions were specifically framed to generate objective fact-based responses.

Information on types of vaccines each company currently produced was sought and number of doses (in millions), of each vaccine supplied in 2018 only, was sought. To ensure accurate and consistent responses the following measures were taken:

1. It was specified that only vaccine types that were bulk manufactured and/or formulated and filled at the manufacturers’ site would be considered. This was to avoid counting errors associated with only secondary packaging or distribution activities.
2. It was specified that the number of doses released in 2018 was sought, not the total manufacturing capacity of their company, nor future capacity.

In regard to research and development, manufactures were asked *to report on vaccine types* they currently had and their respective development stages. To ensure accurate and consistent responses the following measures were taken:

1. Questions specifically targeted vaccine types that were ‘currently’ in the pre-clinical or clinical development stages. Pre-clinical studies had to be active, not in planning stages; vaccine types had to be officially approved to enter the next development stage.
2. Each question regarding the respective development stages were asked separately to avoid any confusion and thus overlapping answers.

To better understand the human resources of the manufacturers, each of the DCVMs were asked to specify number of employees at their company worldwide, the total revenue (in millions of USD) for 2018, and how many countries they supplied with vaccines. In designing these questions we employed the following considerations:

1. The manufacturers were asked to include only formally registered employees, excluding external contracted workers i.e. consultants. Due to the possibility of high rates workforce turnover respondents’ were asked for their best approximation.
2. To enable responses, individual data regarding employees, countries supplied and revenue were agreed to remain confidential.

The methodology used resulted in both quantitative and qualitative responses. The data was then analyzed to produce the findings presented in this report.

The survey was created on the 4th June 2019, and on 6th June 2019 the 43 vaccine manufacturers from the Network were invited by email to respond to the questionnaire. To maximize participation, reminders were sent on the 10th June 2019, 27th June 2019, and 4th September 2019. The survey officially closed on the 30th October 2019 with 34 of 43 DCVMN members formally responding to the survey, resulting in an approximately 80% response rate.

Publicly available information and statistics on varying aspects of company logistics, vaccine production, and vaccine development was compiled from open sources for the 9 DCVMN members who did not formally respond to the survey. This information was included in the results reported here.

Due to sensitivity, not all members were willing to share total revenues, number of employees and number of vaccine doses supplied. As a result the data in this report, in many cases, does not account for all DCVMN members.

## 3 Results: Major achievements

### 3.1 More vaccines from more manufacturers achieving WHO Pre-qualification

The World Health Organisation (WHO) pre-qualification (PQ) programme for vaccines was established in 1987 to respond to the need of procurement agencies and national health agencies to ensure the supply of consistently safe and quality-assured vaccines [8]. Vaccine procurement for UN agencies such as UNICEF continues to be an effective means to ensure the supply of affordable high-quality vaccines globally. In 2016, it was estimated that the WHO enabled vaccine PQ market was valued at 2.1 billion USD [9]. As emerging manufacturers supply more than 50% of the vaccine doses procured by UNICEF, the ability of these manufacturers to meet WHO PQ standards is invaluable.

**Fig. 3:**
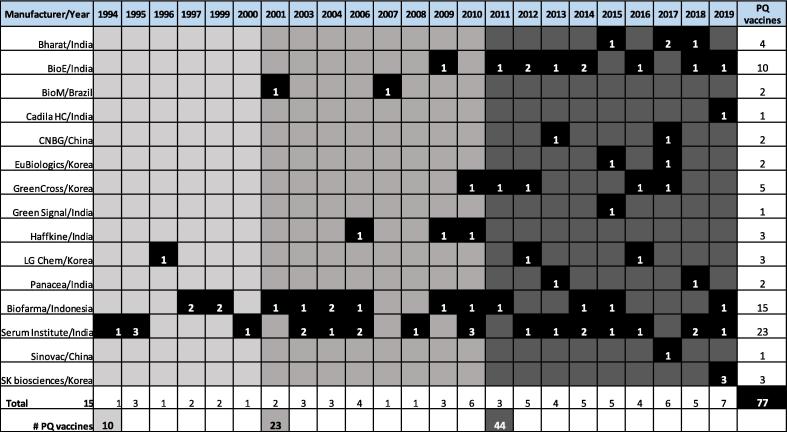
Number of WHO pre-qualified vaccines throughout the years by DCVMN members. Number of WHO pre-qualified vaccines throughout the years by DCVMN members (as of December 31, 2019). Fifteen manufacturers have produced over 70 vaccine types, in various presentations, that have achieved WHO PQ [As of November 1st the freeze-dried BCG vaccine produced by Green Signal Bio Pharma Pvt. Ltd., India was removed from the WHO PQ list. This reduces the number of DCVMN members with PQ vaccines to fourteen.]. The first column lists in name of Network members that have achieved WHO PQ. Each column thereafter represents the year beginning in 1993. Time periods are the illustrated by different shades of grey; 1993–2000 Light grey, 2001–2010 grey, 2011–2019 dark grey. The number within the black square indicates how many vaccines were PQ by the respective manufacturer in the given year. The total number of PQ vaccines from each manufacturer is displayed in the last column, and the aggregate of all WHO PQ vaccines from Network members is displayed in the bottom right corner.

As of October 2019, DCVMN members produced over 70 WHO PQ vaccines types, in a variety of presentations, accounting for half of the total pre-qualified vaccines.^5^ Upon closer examination of timeline of vaccines PQ between 2000 and 2019 (Fig. 3) it is observed that the number of vaccines achieving PQ has doubled in each decade. Notably 30 vaccines from Network members have been prequalified in the past 5 years. Such achievement not only provides an effective mechanism for global vaccine supply but is indicative of the advancements made by developing countries and by manufacturers to meet high standards of manufacturing practices, quality control and regulation. An increasing number of companies manufacturing WHO prequalified vaccines in different countries also serves as a key indicator of the Network achieving its mission.

### 3.2 Contributing to a Polio-free world

The certification of the WHO South East-Asia region as Polio-free in 2014 was a milestone in the Polio Endgame Strategy Plan.^6^ However, outbreaks of circulating vaccine-derived poliovirus (cVDPV) in 2018 [10] and shortcomings in the production of the Inactivated Poliovirus Vaccine (IPV) resulted in 22 countries not being supplied and another 23 experienced serious stock-outs, causing delays in achieving a Polio-free world. Due to the outbreaks of cVDPV the GPEI intends on stopping the use of Oral Poliovirus Vaccine (OPV) once all wild polio virus (WPV) has been eradicated.^7^ Once WPV has been certified as eradicated it is essential that high global coverage with IPV is achieved, so that the world remains polio free [11].

A major breakthrough was the first ever IPV vaccine based on Sabin strains, launched in 2015 by the Institute of Medical Biology Chinese Academy of Medical Sciences (IMBCAMS); unlike the more virulent Salk strain [12], sIPV has lower biosafety risks permitting the manufacturing in developing countries.

The WHO pre-qualification of multidose IPV in 2015, from Bilthoven Biologicals, owned by the Serum Institute of India, enabled UNICEF *offers from more manufactures* for the IPV tender of 2019–2022, and will increase the procurement and access to supply of IPV vaccines [13]. Despite supply issues, IPV has been the fastest new vaccine introduction across countries in the last five years.^8^

Moving forward, the ongoing development of IPV from less-virulent strains, such as Sabin-IPV (sIPV) will be pivotal for the future sustainable supply of IPV.

**Fig. 4:**
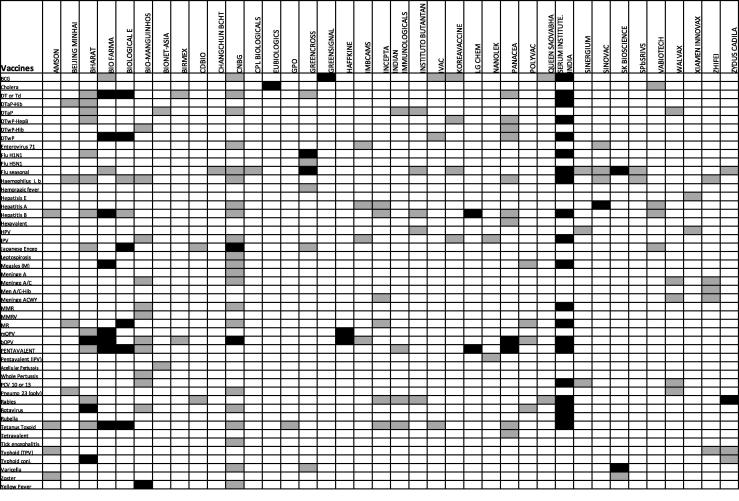
Vaccines produced by DCVMN corporate members as of 2019. Matrix of 196 vaccines produced by 38 DCVMN members (as of October 2019). Of the reported vaccines, 31 manufacturers responded to the DCVMN Progress Report Survey (launched in June 2019), while an additional 7 manufacturer’s vaccine availability report derives from data in the public domain. Vaccine pre-qualified by WHO are indicated in black. Five DCVMN members are currently not producing vaccines. Manufacturers name are as follows: AMSON Vaccines & Pharmaceuticals Ltd., Pakistan; Beijing Minhai Biotechnology Co., Ltd., China; Bharat Biotech International Ltd., India; PT BioFarma, Indonesia; Biological E. Ltd., India; Bio-Manguinhos/Fiocruz, Brazil; BioNet-Asia Co., Ltd., Thailand; Birmex, Laboratorios de Biológico y Reactivos de México, Mexico; CDBIO, Liaoning Chengda Biotechnology, China; Chanhchun BCHT Biotechnology Co., China; CNBG, CNBC consists of 6 Institutes, including National Vaccine and Serum Institute (NVSI), Changchun Institute, Chengdu Institute, Lanzhou Institute, Shanghai, Wuhan, and 2 international trading companies in Beijing; CPL Biologicals Pvt. Ltd., India; Eubiologics Co., Ltd., Republic of Korea; GPO, The Government Pharmeceutical Organization, Thailand; Green Cross, Republic of Korea; Green Signal Bio Pharma Ltd., India; Haffkine Bio-Pharmaceutical Corporation Ltd., India; IMBCAMS, The Institute of Medical Biology, Chinese Academy of Medical Sciences, China; Incepta Pharmaceuticals Ltd., Bangladesh; Indian Immunologicals Ltd., India; Instituto Butantan, Brazil; IVAC, Institute of vaccines and medical biologicals, Vietnam; Korea Vaccine, Republic of Korea; LG Chem, Republic of Korea; Nanolek LLC., Russia; Panacea Biotech Ltd., India; POLYVAC, Center for research and production of vaccines and biologicals, Vietnam; Queen Saovabha Memorial Institute, Thai Red Cross Society, Thailand; Serum Institute of India, India; Sinergium Biotech, Argentina; Sinovac Biotech Ltd., China; SK bioscience, SK Group, Republic of Korea; SPbSRIVS, St. Petersburg Research Institute of Vaccines and Serums and Bacterial preparations plant, Russia; Vabiotech, Company for Vaccine and Biological production No. 1, Vietnam; Walvax Biotechnology Co., Ltd., China; Xiamen Innovax Biotech Co., Ltd., China; ZhiFei Biological Products Co., Ltd., China; Zydus Cadila, India. Biovac Institute, South Africa; BravoVax Co., Ltd., China, Medigen Vaccine Biologics Co., Taiwan; Pasteur Institute of India, India; and VINS Bioproducts Ltd., India are not currently (as of survey responses of October 2019) producing vaccines. The difference between number of PQed vaccines shown in Figs. 3 and 4 is due to the fact that the latter only considers vaccines currently being produced (as to our survey question) while Fig. 3 indicates when these vaccines were pre-qualified but does not mean they are currently being produced. *Note *postscript*: PCV 10 vaccine by Serum Institute received WHO PQ on 17th December 2019. cf. https://extranet.who.int/gavi/PQ_Web/PreviewVaccine.aspx?nav=0&ID=384; bivalent HPV vaccine from Xiamen Innovax approved by Chinese NMPA on 2nd January 2020. cf. https://www.sixthtone.com/news/1005031/china-approves-production-of-first-domestic-hpv-vaccine; PCV 13 vaccine from Walvax approved by Chinese NMPA on 3rd January 2020. http://subsites.chinadaily.com.cn/nmpa/2020-01/03/c_445837.htm; Varicella vaccine from SK bioscience received WHO PQ in 9th December 2019. https://extranet.who.int/gavi/PQ_Web/Default.aspx?nav=2. (For interpretation of the references to colour in this figure legend, the reader is referred to the web version of this article.)

Furthermore, in 2017 the first fully liquid Hexavalent vaccine based on whole-cell pertussis, including IPV, was developed by Panacea Biotech of India [14]. The additional protection against poliovirus further increases coverage against infectious disease in developing countries in an efficient and cost effective manner. As of October 2019, DCVMN members combined were supplying eight vaccine types containing IPV (Fig. 4) and an additional twelve poliovirus vaccines are currently in the pipeline.

### 3.3 Ending cholera

Cholera is an extremely debilitating disease causing acute diarrhea. Following the ingestion of contaminated food or water, transmission is closely linked to poor sanitation and inadequate access to clean water leading Cholera to be endemic in many developing countries.^9^ It has been estimated that there are up to 4 million cases of cholera each year which result in up to 143,000 deaths worldwide [15]. In 1997, a product development partnership between Vabiotech of Vietnam and the International Vaccine Institute (IVI) was the first step towards a safe, effective and affordable cholera vaccine. Vabiotech reformulated their monovalent cholera vaccine and developed the first bivalent oral cholera vaccine (OCV). High yields and low production cost generated a more affordable vaccine [16]. IVI facilitated a technology transfer to a manufacturer in India, to ensure the vaccine could be supplied internationally. Upon pre-qualification in 2011, it became the first affordable OCV vaccine readily available for global supply. Nevertheless, with demand exceeding global supply due to capacity restraints and due to programmatically unsuitable presentation [17], IVI, motivated to increase OCV global supply, formed a partnership with EuBiologics, of South Korea, to produce an optimized OCV product with an innovative plastic presentation, programmatically more suitable, enabling higher volume supply and uptake.

Investment from external stakeholders,^10^ and including the DCVMN member Green Cross Corporation and IVI’s sharing of best practices helped rapidly advance the development, clinical trials and registration of the vaccine. In December 2015 it had achieved WHO pre-qualification.^5^ The vaccine is one of two products available for mass vaccination campaigns through the Global OCV Stockpile and its ongoing production will be vital in achieving the WHO objective of reducing Cholera deaths by 90% by 2030 [18]. Eubiologics followed on from this achievement by producing the innovative first oral cholera vaccine available in plastic vials, reducing the secondary package volume by 30% and weight by 50%, facilitating shipping and improving supply. After achieving WHO prequalification in August 2017, the more cost-effective vaccine doses, in their innovative presentations, were easier to distribute and administer, improving supply and ultimately reducing the burden of endemic cholera. The story of oral cholera vaccines illustrates the importance of technology transfer, collaboration and sharing of best practices for developing country manufactures. Such interactions between manufactures and international bodies in addition to innovation are key factors driving the progress of DCVMN members.

### 3.4 Tackling re-emerging diseases

The measles vaccines have prevented an estimated 23.2 million deaths between 2000 and 2018.^11^ Despite safe and affordable vaccines being available, there has been a three-fold increase in measles cases globally between 2018 and 2019 with not only developing countries being affected; the United States has experienced their highest rates of measles in 25 years [19]. Causes of such outbreaks include fragile pockets of low coverage, skepticism on vaccines and the highly contagious nature of the disease. Network manufacturers continue to play a role with seven company members currently producing measles containing vaccines (MCV) (Fig. 4). Most recently a Measles & Rubella vaccine from Biological E achieved WHO PQ, in October 2019^5^.

Pentavalent vaccines protect children against five major diseases: diphtheria, tetanus, pertussis, hepatitis B and *Haemophilus influenza type b*. With Gavi’s support by the end of 2017 over 404 million children were immunized with pentavalent vaccines [20]. This has not only improved coverage against life-threatening diseases but also reduced the number of injections children receive and the environmental impact associated with disposal of syringes [21]. The first fully liquid pentavalent (DTwPHepBHib) as well as the first fully liquid hexavalent vaccine, based on whole-cell pertussis vaccine, were developed, manufactured and launched by Panacea Biotec in India in 2008 and 2017, respectively. Currently seven Network members produce pentavalent vaccines, five of which are prequalified by WHO (Fig. 4). UNICEF demand forecast for pentavalent vaccines for 2017–2019 was 449 million doses, for which five of the seven suppliers are DCVMN manufacturers [22].

### 3.5 Track-record of vaccine innovations

The first meningitis A vaccine developed specifically for use in Africa was manufactured by Serum Institute of India and prequalified by WHO in 2010, leading the charge to eliminate meningococcal epidemics from the African meningitis belt countries [23].

Another successful innovation came from the development of the world’s first Typhoid Conjugate Vaccine (TCV) by Bharat Biotech International, launched in 2013 [24]. This novel vaccine received prequalification from WHO in 2018 enabling procurement for UNICEF, PAHO and Gavi-supported countries. Bharat is committed to supplying an affordable vaccine globally for GAVI supported countries and is actively working to increase manufacturing capacity to increase supply fourfold [25]. The development and prequalification of TCV is a milestone achievement in the fight to reduce the regional burden of infectious disease.

In 2017, the Serum Institute of India developed the first thermostable rotavirus vaccine. This vaccine innovation generated great benefits, in particular in low income countries where poor infrastructure and frequent disruptions in electricity may compromise the cold chain. The heat stable characteristics of the vaccine ensure it can be safely stored in such circumstances [26]. Rotaviruses are the most common cause of severe diarrheal among young children worldwide, resulting in 215,000 deaths of children under the age of five according to WHO estimates.^12^ Due to the severity of this disease, rotavirus vaccines should be a priority for all national immunization programs globally. The WHO prequalification of rotavirus vaccines (also available from Bharat Biotech) in 2018 will help expand global supply and induce greater market competition [27].

In 2015 and 2016, the first and second EV-71 vaccines against hand-foot and mouth disease were developed by IMBCAMS and Sinovac, and launched in China [28] to fight the emerging disease, mostly in South East Asia.

Currently, 15 licensed influenza vaccines are available from DCVMN members in various countries/regions and 11 vaccines are against seasonal influenza (see Fig. 4). Notably, both Quadrivalent and Trivalent influenza vaccines from SK bioscience were prequalified by WHO in 2019, making them the first WHO prequalified cell-culture based influenza vaccines in the world.

Rabies is a vaccine-preventable zoonotic viral disease, causing about 60,000 deaths annually, over 40% are children under the age of 15, mainly in rural areas of economically disadvantaged countries in Africa and Asia.^13^ A novel rabies vaccine by Zydus Cadila achieved WHO PQ in February 2019. The novel rabies post-exposure prophylaxis consists of a cocktail of monoclonal antibodies combined with the rabies vaccine.^14^ Each year, more than 29 million worldwide receive post-bite vaccination, estimated to prevent hundreds of thousands of rabies deaths, thus WHO has launched a Global Strategic Plan to eliminate human deaths from dog-transmitted rabies by 2030, in which the first objective is to effectively respond through the use of vaccines [29].

### 4 Accelerating the R&D Pipeline

Importantly, ongoing commitment to R&D of vaccines to improve existing vaccines and to prevent known and emerging infectious diseases is needed. R&D efforts will increase the development of next generation vaccines for diseases such as malaria, dengue and tuberculosis (TB), and will lead to improving their attributes towards greater acceptability/cost effectiveness and to the production of vaccines in the regions where they are needed. This survey was also tailored to better understand commitments to R&D and the capacity of manufacturers to advance vaccine projects.

**Fig. 5:**
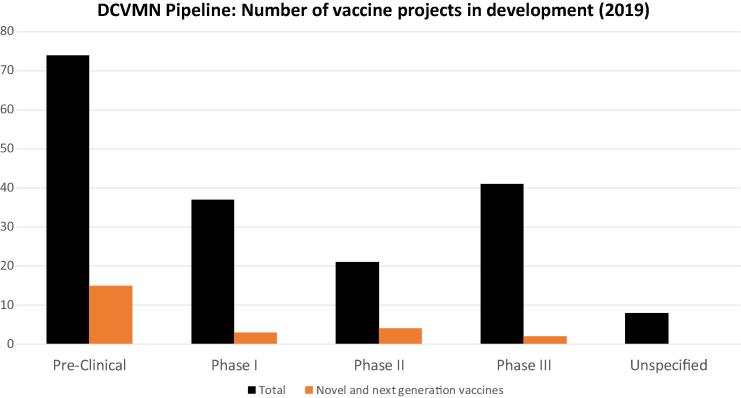
DCVMN members’ pipeline. Number of vaccine types DCVMN members have in research and development (R&D) in the various stages (as of October 2019). Twenty-eight manufacturers shared specifically with stage of R&D their vaccines were in, while five manufacturers reported that they are currently not developing vaccines. Data is derived from the DCVMN Progress Report survey (launched in June 2019). Outcomes of the nine manufacturers who did not respond to the survey are as follows; five publicly shared the stages of the vaccine projects hence the data is included above; two manufacturers shared which vaccine types they are developing but do not specify in which stage therefore these eight vaccine projects are ‘Unspecified’, two manufacturers publicly shared they not are not currently developing vaccines. Black columns indicate the total number of vaccine types with the orange columns corresponding to how many of these vaccines are considered novel. Novel and next generation vaccines include: Chikungunya, Dengue, Malaria, Zika, RSV, GBS, Men ABCWY, Men ABWYX, 4-valent HFMD, HIV, HSV, Shigella, Non-Typhoid Salmonella conjugate, *SalmonellaParatyphi*.

As of October 2019, Network members had 181 vaccine projects in the R&D pipeline, 24 of which are novel vaccines never licensed, such as chikungunya, Zika, RSV (respiratory syncitial virus), HIV or second generation vaccines against dengue and malaria (Fig. 5). Notable, 41 vaccine projects are currently in Phase III clinical trials of which two are considered novel. This marks a significant increase from similar data from 2011 [30], which indicated members had 11 vaccines in late development stage. This illustrates DCVMs growing capacity and willingness to effectively advance their vaccine clinical development. Network members are actively developing over fifty vaccine types to fight against global and regional infectious diseases.

#### 4.1 Novel and next generation vaccines against mosquito-borne diseases

Dengue, a mosquito-borne viral disease, has had a significant increase in global incidence in the last 50 years with about 3.9 billion people, in 138 countries being at risk of infection with dengue viruses.^15^ Predominantly affecting the regions of the Americas, South-East Asia and Western Pacific, an estimated 390 million dengue infections occur annually with 500,000 of these resulting in hospitalizations [31]. Despite the severity and magnitude of the disease, only one vaccine has been licensed to date, however its protective value is limited to persons who have previously had dengue fever [32]. Protection for naïve subjects is needed, thus currently five Network manufacturers have next generation dengue vaccine candidates in the pipeline. The most advanced is a tetravalent vaccine from Butantan Institute, currently in Phase III trials in Brazil. The development of this vaccine began in collaboration with the United States National Institutes of Health (NIH) who transferred their live attenuated dengue vaccine technology to the Butantan Institute [33]. A live attenuated dengue vaccine that induces both humoral and cellular immune responses, is expected to provide lifelong immunity, requiring only one or two doses. Butantan Institute has recruited 15,500 participants who will be monitored for five years to prove the vaccine’s efficacy against all four dengue virus serotypes. Moreover, Butantan entered a research collaboration with a US based company, generating a platform to share data and practices to help advance clinical trials of other dengue vaccine candidates [34]. This collaboration formed on the basis that both dengue candidate vaccines, from Butantan and from a US-based company, are based on a set of genetically modified dengue virus constructs, developed at the US NIH, and provides opportunity to supply dengue vaccines in both a public and private markets.

The development of Butantan’s dengue vaccine illustrates an outstanding commitment to innovation as a tool to fight infectious diseases but also the need for collaboration between academic institutions and vaccine manufacturers.

Another, mosquito-borne disease, the Zika virus infection, has generally mild symptoms such as fever, rash and muscle pain; however infection during pregnancy can have significant consequences of fetal loss, stillbirth and microcephaly.^16^ Worldwide 86 countries and territories have reported evidence of mosquito-transmitted Zika infection, yet no vaccines are currently available, making the development of Zika vaccines a priority for WHO, as the disease is a global health concern and requires a coordinated response [35]. As of October 2019, four Network members had Zika vaccine projects in the research and development stages. The most advanced is a Vero-cell derived inactivated vaccine from Bharat Biotech, which was scheduled to enter Phase II clinical trials at the end of 2019 [36].

Technology platforms used to develop Zika vaccines have also been used in the development of vaccines against chikungunya, another viral disease transmitted by mosquitos causing fever and severe joint pain.^17^ As over one billion people live in areas where chikungunya is endemic and the societal cost of the disease is estimated to be $185 billion USD in the Americas alone, chikungunya has been listed as a priority disease by CEPI.^18^ Low funding and lack of awareness of the disease has slowed the advancement of chikungunya vaccine candidates; therefore there is a need to bring stakeholders together to explore R&D opportunities and to move the pipeline forward [37].

Malaria is another mosquito-borne infectious disease that requires innovation to develop more effective vaccines, as improvements to the low efficacy of the currently available RTS,S/AS01 malaria vaccine is needed.^19^ In 2017, there were an estimated 219 million cases and 435,000 malaria related deaths making it the sixth most deadly disease in low income countries [38]. To contribute to the development and production of next generation malaria vaccines, DCVMs will likely seek collaborations with academic and international organizations. Oxford University’s Jenner Institute has developed a malaria vaccine candidate by using chimpanzee adenovirus vectors, which showed promising Phase II trial results with the ChAd63 ME-TRAP vaccine against infection with *Plasmodium falciparum*, one of the parasites type to cause malaria, supporting the safety of vectored vaccines [39]. VaxHub,^20^ a research based collaboration between the Jenner Institute and University College London Biochemical Engineering along with five ‘spokes’ (including DCVMN members PT Biofarma and Serum Institute of India), is a mechanism for transfer of novel vaccine technology platforms such as the adenovirus vectors to DCVMs to enhance vaccine development processes [40].

#### 4.2 Enabling healthier future for girls and women

Human papillomavirus (HPV) is the most common viral infection of the human reproductive system and can cause cervical cancer in women when untreated, which in 2012 resulted in an estimated 266,000 deaths, accounting for 8% of all female cancer related deaths that year [41]. In particular, HPV-16 and HPV-18 are responsible for 71% of cervical cancer cases globally [42]. Quadrivalent, bivalent and nonavalent HPV vaccines, based on recombinant DNA technology have been made commercially available. As of March 2017, 71 countries had introduced HPV vaccines for their national immunization for girls.^21^ Between 2013 and 2017 UNICEF procured 10.8 million doses of HPV vaccines, from two manufacturers for 36 Gavi-eligible countries, however UNICEF anticipates the demand to be approximately 32 million doses per year over 2020–2023 depending on supply availability [43]. The increase in demand is partially due to increased interest in using HPV vaccines in national immunization programs and a result of WHO’s global call to end cervical cancer [44]. Following the successful launch of the first ever commercially available Hepatitis E vaccine in 2012, Xiamen Innovax Biotech of China has published the results of their randomized clinical trial for a bivalent HPV 16/18 vaccine protecting women against the two most cancerous forms of HPV, indicating no adverse effects or negative outcomes associated with pregnancy and high efficacy [45]. Their HPV vaccine was approved on 30th December 2019 for use in China.^22^ As of October 2019, four HPV vaccines from Network members were undergoing Phase III clinical trials. Such efforts from DCVMN manufacturers will contribute to sustainable supply of HPV vaccines and the reduction of cervical cancer globally, enabling many girls and women in developing countries to have a healthier future.

#### 4.3 Improving competitiveness through product innovation and more suppliers

Pneumococcal infections are the most common cause of bacterial pneumonia in children, a disease which is accountable for 15% of all deaths of children under the age of five.^23^ As of 2018, only two manufacturers had WHO prequalified Pneumococcal Conjugated Vaccines (PCV’s), which provided coverage for 70–82% of childhood pneumococcal disease cases in Africa, Asia, Latin America, the Caribbean, and the Pacific [46]. In 2017 UNICEF procured 157 million doses of PCV. UNICEF estimates that demand could increase to 258 million doses by 2027 with one billion doses, required between 2021 and 2027, to immunize 361 million children. This projected increase in demand called for greater responsibility in vaccine supply. Four Network members: Serum Institute of India, Walvax and Beijing Minhai of China, and Nanolek of Russia have reported PCV Phase III clinical trials in this survey. Biological E and other manufacturers are developing PCV vaccines to protect against existing pneumococcal serotypes and emerging novel pneumococcal strains. On December 18, 2019 Serum Institute of India became the first DCVMN member manufacturer to have a PCV prequalified by WHO^5^. This vaccine, made using novel conjugation technology, protects against ten serotypes that are most likely to cause disease in Asia and Africa, areas where the disease is of greatest burden, providing advantages over previous PCV vaccines. Additionally, in late December 2019 China approved the first self-developed 13-valent PCV.^24^ Commitment to innovation provides a powerful tool to reduce the global burden of this infection and highlights the importance of product diversity and industry competition to maximize access and minimize risks in vaccine manufacturing and increase security of supply.

#### 4.4 The unfinished vaccine R&D agenda

Tuberculosis (TB) remains one of the biggest public health challenges, with ten million new cases each year and 1.5 million TB-related deaths in 2018.^25^ Furthermore, the increasing antibiotic resistant strains of *Mycobacterium tuberculosis* (Mtb) exacerbate disease burden, causing high treatment costs and growing fatality rate. There is an urgent need for efficacious vaccines that protect against different forms of TB disease, in various age groups, to fight the global TB epidemic. The Bacillus Calmette-Guérin (BCG) vaccine, developed in 1921, is currently the only licensed vaccine against TB and is manufactured by seven members of the Network. Its use in national immunization programmes has over 80% coverage in neonates and infants, providing good protection against meningitis and disseminated TB in children. However, BCG limitations include variable protection against pulmonary TB and lack of protection against primary infection and reactivation of latent pulmonary infection, the principal source of bacillary spread in the community. In 2018, two clinical trials provided promising results [47]. A robust portfolio and diverse vaccine pipeline together with collaborative research and development are essential to combat TB.

Human immunodeficiency virus (HIV) is one of leading causes of death with an estimated 770,000 deaths from HIV-related illnesses worldwide in 2018.^26^ Challenges in HIV vaccine development are related to the fast mutation rates of the virus resulting in great genetic diversity. Hence in a single individual and within the population there are a significantly large number of replicating viruses, each having a different genetic makeup, so a successful HIV vaccine will have to be able to provide protection against many virus strains [48]. HIV vaccine trials in Thailand showed 31.2% efficacy [49] which was considered an achievement in 2009. Results from ongoing clinical trials in Africa will provide more information on the efficacy and timeline of novel HIV vaccine candidates with the potential for a HIV vaccine to be available by 2030. Currently, just one DCVMN member has reported a HIV vaccine in the R&D pipeline, perhaps due to the high risks associated with this formidable vaccine challenge.

## 5 Conclusion and perspectives

Infectious diseases can spread through communities like wildfire, affecting millions of lives and exacting economic and social costs. Innovation will be central to the success of vaccine manufacturers and advances have been made in recent years, with more vaccines achieving WHO prequalification and novel vaccine types progressing successfully through clinical development towards late clinical trial phases. Having more vaccine manufacturers with the technology, resources and willingness to develop and produce a variety of vaccines in the regions where the incidence of disease is important for prevention strategies.

In the last decade, DCVMN members supplied most of the affordable pentavalent and other combination vaccines, decreasing the gap between high-income and developing countries in DTP3 vaccination coverage from 15% to 10% [5]. Novel vaccines such as rotavirus, pneumococcal, Japanese encephalitis (JE), typhoid, cholera, meningococcal, HPV, and hand-foot and mouth disease, were licensed, launched, or prequalified by WHO. Further, vaccines against dengue, Zika, chikungunya and malaria, are in the pipeline to better protect low-income populations from mosquito-borne diseases and premature mortality. The mission of the Network, to enable supply of high-quality vaccines to all people where most needed, in a sustainable manner, is heading in the right direction, rendering vaccines as a universal good. With 80 percent of the world’s populations living in developing countries it would be appropriate for 80 percent of the vaccines to be produced there, readily available for people of developing countries.

As illustrated above, collaboration, sharing of best practices and technical cooperation has helped shape the last decade of vaccines, by enhancing the ability of manufacturers to supply vaccines where they are needed most. Novel manufacturing technologies and platforms, such as RNA based-vaccines, adenoviral vectors, and bioconjugation as well as novel packaging technologies such as blow-fill-seal, and two-dimensional barcodes provide a fertile ground to accelerate development and access to vaccine innovations. In bringing together vaccine manufacturers in developing countries and international stakeholders, in supporting human resources training in cGMP, QMS, surveillance tools for vaccine safety performance, in facilitating understanding of regulatory systems, in fostering new technologies through academic collaborations and in encouraging WHO prequalification, DCVMN will play an important role in increasing the viability [50] of emerging vaccine manufacturers to innovating as we enter the next decade.

## Declaration of Competing Interest

The authors declare that they have no known competing financial interests or personal relationships that could have appeared to influence the work reported in this paper.

## Footnotes

1 INTERNATIONAL COOPERATION FOR DEVELOPMENT cf. https://www.unsystem.org/content/international-cooperation-development.

2 www.dcvmn.org/resource-members.

3 WHO Immunization coverage factsheet. Available at https://www.who.int/news-room/fact-sheets/detail/immunization-coverage.

4 WHO Global and regional immunization profile. Available at https://www.who.int/immunization/monitoring_surveillance/data/gs_gloprofile.pdf?ua=1.

5 Total pre-qualified vaccines. https://extranet.who.int/gavi/PQ_Web/.

6 Global Polio Eradication Initiative. http://polioeradication.org/.

7 GPEI Vaccine-derived poliovirus factsheet. http://polioeradication.org/wp-content/uploads/2018/07/GPEI-cVDPV-Fact-Sheet-20191115.pdf.

8 https://www.unicef.org/supply/files/Session_13_Presentation_3_IPV_Supply_and_Demand_Update.pdf.

9 Cholera FactSheet. https://www.who.int/news-room/fact-sheets/detail/cholera.

10 http://www.ghif.com/uncategorized/press-release-eubiologics/.

11 Measles Fact Sheet. https://www.who.int/news-room/fact-sheets/detail/measles.

12 Rotavirus. https://www.who.int/immunization/diseases/rotavirus/en/.

13 Rabies Fact sheet. https://www.who.int/news-room/fact-sheets/detail/rabies.

14 https://www.dcvmn.org/IMG/pdf/zydus_receives_who_pq_for_vaxirab_n_-_final_edit.pdf.

15 Dengue Fact sheet. https://www.who.int/news-room/fact-sheets/detail/dengue-and-severe-dengue.

16 Zika Virus Fact Sheet. https://www.who.int/en/news-room/fact-sheets/detail/zika-virus.

17 Chikungunya Fact sheet. https://www.who.int/news-room/fact-sheets/detail/chikungunya.

18 Coalition for Epidemic Preparedness Innovations.

19 https://www.who.int/malaria/media/malaria-vaccine-implementation-qa/en/.

20 Vax Hub. The Future Vaccine Manufacturing Research Hub: securing the supply of essential vaccines. Available at https://www.ucl.ac.uk/biochemical-engineering/research/research-and-training-centres/vax-hub.

21 WHO/Immunization, Vaccines and Biologicals database, as of 31 March 2017. Available at http://www.who.int/entity/immunization/monitoring_surveillance/VaccineIntroStatus.pptx, accessed February 2017.

22 China's domestically produced HPV vaccine gets sale approval from authority. Cf. https://news.cgtn.com/news/2020–01-01/China-s-domestically-produced-HPV-vaccine-gets-official-sale-approval--MTB6oXrWHm/index.html.

23 Pneumoccocal Fact Sheet. https://www.path.org/resources/fact-sheet-pneumococcal-disease-pneumococcal-conjugate-vaccines-and-pneumosil/.

24 http://subsites.chinadaily.com.cn/nmpa/2020–01/03/c_445837.htm & https://www.reuters.com/article/us-walvax-vaccine/china-approves-home-grown-vaccines-offering-alternative-to-global-drugmakers-products-idUSKBN1YZ0JV.

25 https://www.who.int/news-room/fact-sheets/detail/tuberculosis.

26 HIV/AIDS Fact sheet. https://www.who.int/news-room/fact-sheets/detail/hiv-aids.
